# Does Reconstruction with Reimplantation of Sterilized Tumor Bone Provide Survival Benefit in Diaphyseal Osteosarcoma?

**DOI:** 10.1155/2020/4092790

**Published:** 2020-01-19

**Authors:** Prakash Nayak, Ashish Gulia, Ajay Puri

**Affiliations:** Department of Surgical Oncology, Bone and Soft Tissue Disease Management Group, Tata Memorial Centre, Homi Bhabha National Institute, Parel, Mumbai 400012, India

## Abstract

Does reimplantation of sterilized tumor bone for reconstruction provide outcome benefits in intercalary osteosarcoma based on the potential immunogenic effect of reimplanted sterilized tumor tissue? Of 720 cases of surgically treated high-grade osteosarcoma patients treated at our institute from 2006 to 2013, 61 had predominantly diaphyseal disease. All patients were nonmetastatic at presentation. Patient and tumor characteristics, treatment details, and local recurrence-free, metastasis-free, and overall survival were compared for 24 patients who had reconstruction with sterilized tumor bone reimplantation vs 37 who did not. Both the groups were well matched in terms of baseline characteristics. Means were compared with the *t*-test, proportions with the chi-square test, and survival with the log-rank test. The Kaplan‐Meier method was used to construct time to event curves. Cox proportional hazard regression modeling was employed for multivariate time to event analysis. Twenty-two had extracorporeal radiation and reimplantation (ECRT) with or without the vascularised fibula. Fifty-gray single dose was used in all cases. Two had pasteurization and reimplantation. Thirty seven had non-reimplantation reconstructions (including intercalary or osteoarticular endoprosthesis, pedicled bone grafts, rotation-plasty, and amputations). Five-year local recurrence-free survival was 85% for reimplantation and 97% for non-reimplantation groups (*p*=0.17). Five-year metastasis-free survival was 63% and 54%, respectively (*p*=0.44). Five-year overall survival was 70% and 58%, respectively (*p*=0.39). The data from this study did not demonstrate significantly better local recurrence-free, distant relapse-free, or overall survival benefit in the tumor bone reimplantation group.

## 1. Introduction

Limb salvage surgery in extremity sarcoma management is the standard of care. Biologic reconstruction when possible is an attractive and durable alternative to endoprosthesis. It is widely used in diaphyseal sarcomas where saving the adjacent joints is oncologically safe. Reconstruction is usually performed using allografts with or without a live vascularised fibula, diaphyseal endoprosthesis, or reimplantation of the excised bone after sterilizing it using either external radiation [1, 2], pasteurization [3], autoclaving, or liquid nitrogen [4]. Reimplantation offers the advantage of an easier fit to the host bone compared to allografts and obviates the need of a tissue bank. Though the potential risk of local recurrence was of concern earlier, several long term studies have refuted that concern [1, 2, 5]. While there have been some preclinical experimental studies that suggested the potential immunogenic effect of treated tumor tissue reimplantation after cryotherapy where the host is exposed to denatured tumor antigens [5, 6], clinical data for validating this hypothesis in reimplanted sterilized tumor bone are lacking. We sought to determine if reimplanting sterilized tumor bone (extracorporeal radiation and reimplantation (ECRT)/pasteurization) provides any survival benefit over other forms of reconstructions in diaphyseal osteosarcoma.

## 2. Methods

### 2.1. Patients

All consecutive diaphyseal osteosarcoma patients operated between January 2006 and December 2013 at our institution were included in the study. Patient details were identified from a prospectively maintained database. Patient and tumor characteristics, treatment details, and local recurrence-free, metastasis-free, and overall survival were retrieved. Prior to surgery all patients underwent staging investigations which included plain radiographs and MRI of the limb, CT scan of the chest, and a bone scan. All patients were nonmetastatic at presentation. They received neoadjuvant and adjuvant chemotherapy as per the existing hospital protocol [7]. Sixty one of 720(8.4%) patients had diaphyseal disease. The median age of the cohort was 17 years (range 6 to 58 years), with 47 males and 14 females. Twenty-four patients (39%) had reimplantation of tumor bone 22 ECRT and 2 pasteurized tumor bone (as part of a clinical trial) [3]. Of the remaining 37 patients, 4 (6%) had an amputation, 4 (6%) had allograft reconstruction, 7 (11%) had vascularised bone transfers, 3 (5%) had cement spacers, 1 (2%) rotation-plasty, and 18 (29%) had endoprosthetic reconstructions.

Reimplanting sterilized tumor bone is not advisable in tumor bones which are structurally weak and in bones with pathological fractures. In such cases, alternative means of reconstruction were used. In cases where the diaphyseal tumors extended very close to the articular surface and the osteoarticular part of the bone necessitated resection for oncologic reasons, endoprosthetic reconstructions were used [2].

Our surgical technique and method of ECRT has been documented in detail earlier [2]. A 2 to 3 cm marrow margin as calculated on the T1-weighted MRI image was considered an adequate resection margin.

After tumor excision, a sample of the marrow was sent for a frozen section from both residual ends of the host bone, to confirm clear margins. The resected specimen was then transferred to a separate sterile trolley, away from the main operative field to avoid any contamination of the operative field. Under aseptic precautions, all the soft tissue including the periosteum was stripped from the bone after inking the closest soft tissue margin. This inking of margins helped the pathologist report on the adequacy of resection in the final histopathology report, which otherwise would not have been possible [2].

The resected bone was irradiated to a dose 50 Gy/1 fraction prescribed to the midplane of the specimen using 6 MV photons or 60 Cobalt gamma rays with parallel opposing portals. On returning to the operative room, the marrow contents were reamed and the bone specimen was lavaged with a high-speed pulsatile lavage system to remove residual marrow tissue. Bone cement was packed in the medullary cavity of the radiated graft, and the specimen was realigned with the host bone and stabilised with suitable internal fixation. We thus sought to compare 24 cases of reimplanted tumor bone with 37 cases treated without reimplantation. Both the groups were well matched in terms of baseline characteristics except marked male preponderance in the reimplantation of the tumor group (Table 1). All patients in both groups had a negative margin resection. None of the patients received postoperative radiotherapy. Postprocedure surveillance included a follow-up visit every 3 months for the first 2 years and 6 monthly subsequently.

### 2.2. Statistical Analyses

Statistical analyses were completed in R version 3.2.2, including the survminer package from the Comprehensive R Archive Network. Time to local relapse, systemic relapse, and death from any cause were calculated from the date of surgery. We defined recurrence as clinical, radiological, or pathological evidence at primary or metastatic site. Patients who did not have an event at the end of study duration were censored at the date of last follow-up, death from disease, or other causes. The Kaplan‐Meier method was used to construct time to event curves. Cox proportional hazard regression modeling was employed for multivariate time to event analysis with significance set at *p* < 0.05.

## 3. Results

**Figure 1:**
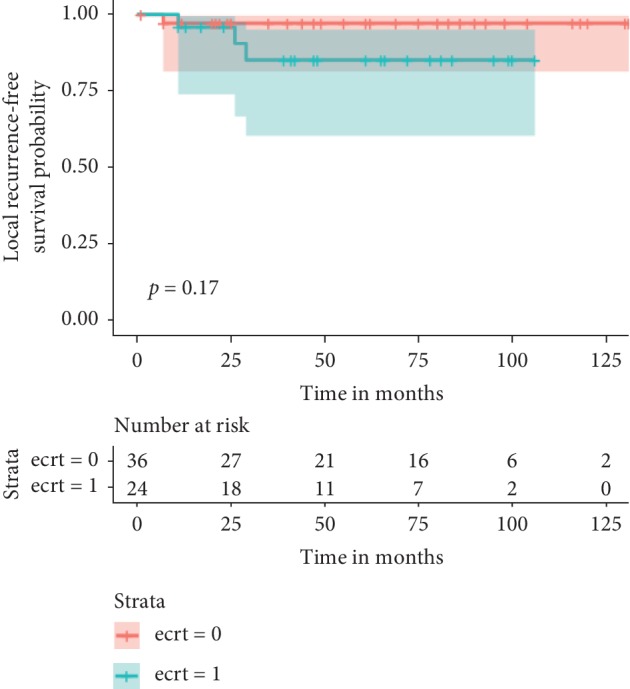
Kaplan‐Meier curves showing local recurrence-free survival stratified by use of reimplantation. Green line with reimplantation of tumor bone and the red without. Time is in months.

**Figure 2:**
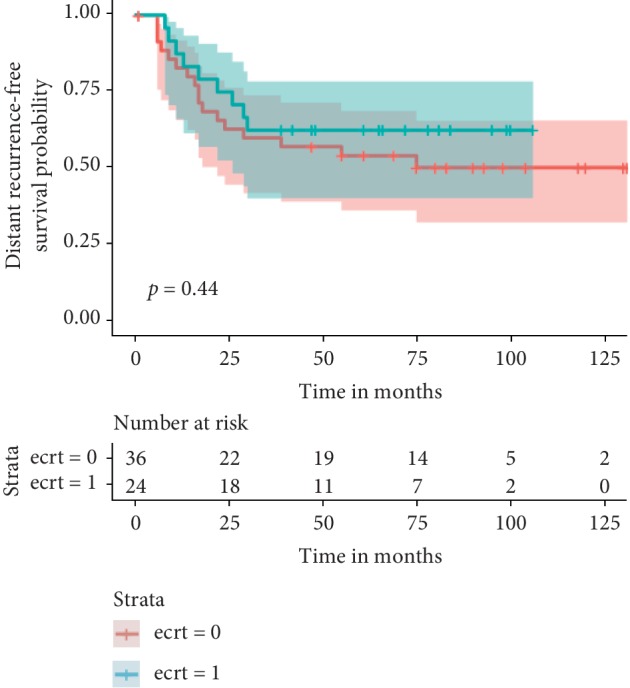
Kaplan‐Meier curves showing distant recurrence-free survival stratified by use of reimplantation. Green line with reimplantation of tumor bone and the red without. Time is in months.

**Figure 3:**
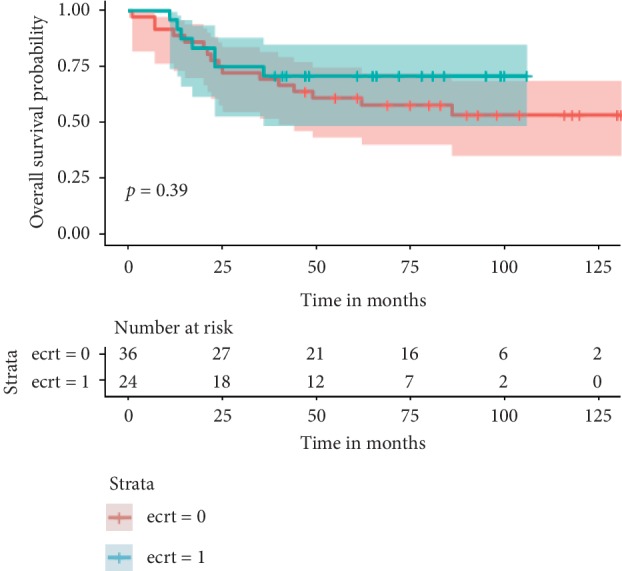
Kaplan‐Meier curves showing overall survival stratified by use of reimplantation. Green line with reimplantation of tumor bone and the red without. Time is in months.

The median follow-up in survivors was 80 months (range 15 to 131) and in those who died of disease was 22 months (range 1 to 86). The median follow-up in the reimplantation group was 54 months (range 11–106) and in the non-reimplantation group was 62 months (range 1–131). Twenty-three patients had died at the time of evaluation (7/24 reimplantation; 16/37 non-reimplantation), 5 (2/24 reimplantation; 3/37 non-reimplantation) were alive with disease, and 33 were free of disease (15/24 reimplantation; 18/37 non-reimplantation). Thirty three (54%) remained continually disease-free with a median DFS of 80 months (range 15–131 months). In all 3 local recurrences in the reimplantation group, the recurrence was in the adjacent soft tissue and not in reimplanted bone. The oncologic outcomes of local relapse, systemic relapse, death, and actuarial survival estimates are depicted in the table and figures below (Table 2, Figures 1–3).

## 4. Discussion

The potential advantage of tumor immunogenicity to help improve survival has not been tested as a hypothesis in human study cohorts. Nishida et al. demonstrated the induction of systemic antitumor response in malignant bone tumors following reconstruction with frozen autografts using liquid nitrogen in a murine osteosarcoma model and in a few select human cohorts with unresectable metastatic osteosarcoma combined with dendritic cell therapy [4, 5]. Various other studies have also shown immunological effects of cryosurgery [6, 8]. Some studies postulated that tissue proteins released from frozen lesions may have antigenic properties which may initiate an immune tumor response [9–11].

Extracorporeal radiation and reimplantation was first reported in 1968. The single high dose ensures tumor death avoiding radiation to surrounding normal tissue. Pasteurization (heating at 60–65 degree celsius for 40 min) eradicates tumor cells as shown in both preclinical and clinical studies [3, 12, 13]. It is reasonable to extend the hypothesis of immunological effects of cryosurgery to extracorporeal radiation and reimplantation as well which allows the immune system to access denatured tumor antigens. Demaria et al. demonstrated that the abscopal effect induced by local radiotherapy (regression of nonirradiated metastatic lesions at a distance from the primary site of irradiation) can be considered as a systemic antitumor immune response as it provides both, tumor-specific antigens from dying cells and maturation stimuli for tumor-specific T cells to mediate distant tumor inhibition [14].

We therefore analysed our results from a subset of one of the largest series of osteosarcoma treated at a single institute to test this hypothesis in a clinical setting [7]. The strengths of this study are the uniform decision protocols with controlled heterogeneity, all performed at a tertiary sarcoma centre with consistent treatment protocols. One of the limitations of tumor bone reimplantation is the absence of postchemotherapy tumor necrosis evaluation which to date remains one of the most significant prognostic markers of outcomes in osteosarcoma. We were thus unable to use postchemotherapy tumor necrosis as a comparator between the 2 groups (absent in the reimplanted subset as tumor was sterilized and reimplanted). Since the differences in size of tumor and length of resection at diagnosis between the two groups were non significant and similar chemotherapy protocols were employed, a random distribution of tumor necrosis across the cohort is a reasonable imputation.

Another limitation is the fact that being a retrospective analysis, we have not measured any serum cytokine levels to objectively document an immune response.

Although unavoidable, the decision of choice of reconstruction was done in an observational fashion and was not randomized. This is inevitable as numerous factors including patient choice can influence the decision regarding choice of reconstruction modalities.

Despite these limitations, this is a reasonable comparison of survival possible in a real world scenario. The reimplantation group did not show significantly better local recurrence-free, distant relapse-free, or overall survival benefit in this series though there was a trend to superior overall survival that did not reach statistical significance. A randomised trial after propensity-matched scoring in a cohort with serum cytokine levels is ideal for answering potential causal links between sterilized tumor bone reimplantation and immune mediated survival but unlikely in a real world scenario. Our data thus failed to show a statistically superior survival benefit in the group receiving tumor bone reimplantation.

## Figures and Tables

**Table 1:** Baseline characteristics of reimplantation and the non-reimplantation groups.

Variables	Reimplantation group	Non-reimplantation group	*p* values
*n*	24	37	
Female sex	1 (4%)	13 (35%)	0.01
Median age, year (range)	17 (8–35)	17 (6–58)	
Mean length of resection, cm (range)	20 (10–45)	22 (11–45)	0.07
Proportion of tumors of size >8 cm (%)	80	90	0.3
Median follow-up, month (range)	54 (11–106)	62 (1–131)	

**Table 2:** Summary of outcome variables in the reimplantation and the non-reimplantation group.

Variable	Reimplantation group (*n* = 24)	Non-reimplantation group (*n* = 37)	*p* values
Local recurrence only	1 (4%)	1 (2.7%)	
Metastasis only	6 (25%)	18 (43%)	
Local recurrence with metastases	2 (8%)	0 (0%)	
Died of disease	7 (29%)	16 (43%)	
Alive with disease	2 (8%)	3 (8%)	
Continually disease free	15 (62.5%)	18 (48.64%)	
5 yr LRFS	85% (range 60–90)	97 (range 80–95)	0.17
5 yr MFS	63 (range 40–78)	54 (range 35–70)	0.44
5 yr OS	70 (range 50–85)	58 (range 40–70)	0.39

LRFS: local recurrence-free survival; MFS: metastasis-free survival; OS: overall survival.

## Data Availability

The retrospective data used to support the findings of this study are available from the corresponding author upon request.
